# Association of triglyceride glucose index and triglyceride HDL ratio with glucose levels, microvascular and macrovascular complications in Diabetes Mellitus Type-2

**DOI:** 10.12669/pjms.39.5.7389

**Published:** 2023

**Authors:** Wajiha Mah Jabeen, Basmah Jahangir, Saba Khilji, Aqsa Aslam

**Affiliations:** 1 Wajiha Mah Jabeen, MBBS, Mphil, PhD Professor of Pathology, HITEC-Institute of Medical Sciences, Taxila, National University of Medical Sciences (NUMS), Rawalpindi, Pakistan; 2 Basmah Jahangir, BS MLT Department of medical laboratory Technology, Islamabad Medical and Dental College, Islamabad, Pakistan; 3 Saba Khilji, MBBS Lecturer of Pathology, Islamabad Medical and Dental College, Islamabad, Pakistan; 4 Aqsa Aslam, MBBS, Mphil Assistant Professor of Pathology, Islamabad Medical and Dental College, Islamabad, Pakistan

**Keywords:** Diabetes Mellitus Type-2, Triglyceride glucose index, Triglyceride HDL ratio, Diabetes-associated complications

## Abstract

**Objective:**

To find out the role of triglyceride glucose index (TGI) and triglyceride HDL ratio (THR) as predictors of insulin resistance and control of glucose status in type two diabetes mellitus (T2DM).

**Method:**

This cross-sectional study was conducted in Dr. Akbar Niazi Teaching Hospital, January-April 2022. A total of 56 individuals, both males and females aged 30-75 years having T2DM with fasting blood glucose ≥ 110 mg/dl and HbA1c ≥ 5.7% were included. Biochemical markers were estimated by applying standard methods. Independent sample t-test, Fisher exact test, and linear regression were applied.

**Results:**

TGI and triglyceride HDL ratio were significantly raised (p=0.01) in patients with poor glycemic control as compared to controlled glucose levels (17.8 ± 4.7vs7.3 ± 1.75) and (3.84 ± 1.3vs2.12 ± 0.64) respectively. These two indices have a significant association (p=0.01) with HbA1c (r=0.963, r=0.757), fasting blood glucose (r=0.964, r=0.748), and HOMA-IR (r=0.955, r=0.718) respectively. Moreover, TGI and THR were found to have a more significant association with the development of cardiovascular disease (CVD) (r=0.717, r=0.555) and a significant but weak association with nephropathy (r=0.385, r=0.302) respectively. Regression analysis revealed that both TGI and THR have significant predictive ability for HbA1c, fasting blood glucose, HOMA-IR and CVD (delta R^2^=0.738vs 0.408, 0.740vs0.395, 0.725vs0.362, 0.354vs0.170) respectively, after controlling all confounding variables.

**Conclusions:**

TGI and THR have a strong association and predictive capability to identify insulin resistance and detect the development and progression of T2DM. Moreover, TGI can be more precisely used for prediction analysis as compared to THR.

## INTRODUCTION

Type-2 diabetes mellitus (T2DM) with chronic hyperglycemia can lead to various organ system damage that can cause life-threatening health complications.^1^ The prevalence of T2DM is more in South East Asia (11.3%) in comparison with developed countries.^2^ Specifically in Pakistan, it was estimated to be 11.7% along with a raised incidence of microvascular complications, especially nephropathy, 130.2 per 1000 persons per year.^3^,^4^ Diagnosis and treatment of T2DM are costly and can burden people significantly.^5^ Triglyceride glucose index (TGI) and triglyceride-HDL ratio (THR) have been proposed as indicative and predictive markers of T2DM.

It can be analyzed more frequently in routine clinical practice, as triglyceride, HDL, and glucose levels are routinely performed and their cost is affordable.^6^-^11^ Data indicating the significance of TGI and THR in T2DM is still lacking in Pakistan. That’s why we had planned this study to find out the association and predictive ability of TGI and THR with reference to glucose status and micro & macrovascular complications in T2DM patients.

## METHODS

This cross-sectional study was conducted in Dr. Akbar Niazi Teaching Hospital, January-April 2022. Both males and females aged 30-75 years with FBG ≥ 110 mg/dl and HbA1c ≥ 5.7% were included in the study.

### Inclusion & Exclusion Criteria

Patients with a history of acute illness, thyroid, liver, or malignant disorders, acquired immunodeficiency disease, uncontrolled hypertension (systolic blood pressure (BP) >180 mm/Hg and diastolic BP > 110 mm/Hg), pregnancy or lactation were excluded from the study.

The sample size was calculated with a World Health Organization calculator using a 95% confidence interval, and 80% power of the study. The level of anticipated TGI in Group-1 was 4.94 ± 0.38 and in Group-1 was 5.21 ± 0.34.^10^ In the reference study, mean and standard error mean (SEM) were given. We have converted the SEM into standard deviation (SD) to calculate the sample size. The calculated sample size was 28 patients in each group. Patients who fulfilled the inclusion and exclusion criteria were inducted and divided into Group-1: HbA1c ≤ 7% (n=28); Group-1: HbA1c > 7% (n=28). Informed consent was taken from all the participants.

### Ethical Approval

The study was approved by the ethical committee of Islamabad Medical and Dental College (46/IMDC/IRB-2021, date: 22-12-21). ***Data collection and analysis:*** Demographic data of the patients were collected through a questionnaire. A total of 5 ml blood samples were collected. Biochemical markers were estimated according to the procedures recommended by the manufacturer. The Bio-Rad, D-10 instrument was used to estimate HbA1c levels. Cholesterol, Fasting blood glucose (FBG), triglyceride, HDL and LDL were estimated by Selectra pro-M. Serum Insulin estimation was done on ADVIA^®^ Centaur immunoassay system (USA). HOMA-IR was derived by the formula “Fasting Insulin:

(µU/L) × FBG (mg/dL) / 405. The Following formulas were used to calculate TGI and THR: “TGI = [Serum triglyceride(mg/dL) × FBG(mg/dL) / 2]” and “THR = serum triglyceride(mg/dL)/serum HDL(mg/dL)”.

### Statistical analysis

Statistical Analysis was performed using SPSS version 21. The Kolmogorov-Smirnov (KS) (Please write full name and then abbreviation) test assessed the normality of data. The quantitative and qualitative data were measured as mean± SD and number (percentage) respectively. Independent sample t-test and fisher exact tests were used for the comparison of quantitative and qualitative variables respectively. Pearson correlation was applied to determine the association of TGI and THR with all variables including gender, BMI, age, waist circumference, BP, duration of diabetes, FBG, HbA1C, fasting insulin, HOMA-IR, lipid profile, retinopathy, nephropathy, neuropathy and cardiovascular disease (Please name those variables). Sequential multiple regression was used to assess the predictive ability of TGI and THR. In regression analysis, we have controlled all confounding variables in model 1. In subsequent models, we have added other variables one by one to determine their impact on the models. (Please mention in the methodology how did you adjust for different variables while performing linear regression analysis in different models). P-value <0.05 was considered statistically significant.

## RESULTS

A total of 56 patients; 37 (66%) males and 19 (34%) females participated in the study. There was no significant difference in the basic demographics of the two groups. Patients of T2DM in the HbA1c > 7% group had markedly higher levels of FBS, fasting insulin, HOMA-IR, TGI, THR and cardiovascular disease (CVD) events, Table-I. TGI and THR both showed a significant positive correlation with BMI, age, blood pressure, duration of analysis, FBS, fasting insulin, HbA1c, HOMA-IR, and TG. TGI was significantly positively correlated with nephropathy, neuropathy, and CVD.

THR showed a significant positive association with nephropathy and CVD. High R-values indicate a stronger association of TGI with variables in comparison with THR, Table-II. After controlling the confounding variables in model-1, the addition of HbA1c in model-2 shows a significant amount of variance. It indicates the strong significant predictive ability of TGI and THR for HbA1c. The addition of FPG, HOMA-IR and CVD in models-3,4 and 5 slightly increases the model’s strength, Table-III. Independent models reveal the significant separate predictive ability of TGI and THR for HbA1c, FPG, HOMA-IR and CVD in models-2, 3, 4 and 5 respectively, Table-IV.

**Table-I:** Comparison of demographic and biochemical variables in patients with T2DM.

Variable	HbA1c ≤ 7 (n=28) Mean ± SD	HbA1c >7 (n=28) Mean ± SD	*P-value
Age (years)	57.86 ± 9.60	55.56 ± 8.4	0.342
Weight (kg)	79.96 ± 5.63	79.89 ± 3.28	0.950
Height (meter)	1.72 ± 0.07	1.70 ± 0.09	0.372
BMI (kg/m^2^)	26.98 ± 2.04	27.73 ± 3.00	0.285
Waist circumference (inches)	39.59 ± 3.22	39.41 ± 4.56	0.567
Systolic BP (mmHg)	143.45 ± 9.17	140.74 ± 6.46	0.205
Diastolic BP (mmHg)	93.79 ± 6.36	92.96 ± 5.59	0.606
Duration of diabetes (Years)	11.81 ± 4.76	13.17 ±4.50	0.278
FBS (mg/dL)	139.43± 10.16	205.70 ± 23.01	0.001
HbA1c (%)	6.49±0.36	8.82±0.83	0.001
Insulin (µIU/mL)	12.09±0.75	17.77±2.23	0.001
HOMA-IR	4.17±0.49	9.14±2.01	0.001
TG (mg/dL)	168.36± 33.85	272.42 ± 49.65	0.001
Cholesterol (mg/dL)	210.48± 30.72	222.93 ± 23.25	0.095
HDL (mg/dL)	35.90 ± 7.25	33.52 ± 9.03	0.280
LDL (mg/dL)	135.57 ± 30.38	148.94 ± 31.25	0.111
TGI	11795.70 ± 2801.67	28479.70 ± 7509.51	0.001
THR	4.86 ± 1.47	8.81 ± 3.02	0.001

*Complications*	*Number (Percentage)*	*Number (Percentage)*	**P-Value

Retinopathy	1(3.5)	2(7.14)	0.600
Nephropathy	2(7.14)	7(25)	0.087
Neuropathy	0(0)	2(7.14)	0.161
CVD	0(0)	17(60.7)	0.001

* Independent t-test and

** Fischer exact test.

**Table-II:** Correlation of TGI and THR with demographic and biochemical Variables in patients with T2DM.

Variables	TGI		THR	

	R-value	*p-value	R-value	*p-value
Gender	-0.037	0.787	-0.057	0.677
BMI	0.554	0.001	0.329	0.013
Age	0.443	0.001	0.420	0.001
Waist circumference	0.271	0.043	0.222	0.101
Systolic BP	0.731	0.001	0.556	0.001
Diastolic BP	0.679	0.001	0.559	0.001
Duration of Diabetes (years)	0.753	0.001	0.616	0.001
FBS	0.964	0.001	0.748	0.001
HbA1c	0.963	0.001	0.757	0.001
Fasting Insulin	0.929	0.001	0.680	0.001
HOMA-IR	0.955	0.001	0.718	0.001
Cholesterol	0.200	0.139	0.203	0.133
TG	0.977	0.001	0.813	0.001
HDL	-0.266	0.047	-0.689	0.001
LDL	0.233	0.084	0.219	0.105
Retinopathy	0.087	0.525	0.148	0.277
Nephropathy	0.385	0.003	0.302	0.024
Neuropathy	0.290	0.030	0.184	0.176
Cardiovascular disease	0.717	0.001	0.555	0.001

* Pearson’s Correlation.

**Table-III:** Regression analysis models show the predictive ability of TGI and THR for biochemical variables and diabetes-associated complication

Predictors	TGI^a^			THR^b^		

	*Beta	*R^2^	*Delta R^2^	*Beta	*R^2^	*Delta R^2^
Model-1		0.201			0.232	
Model-2	4.674^***^	0.939	0.738^***^	0.737^***^	0.640	0.408^***^
Model-3	2.432	0.941	0.002	-1.587	0.658	0.019
Model-4	0.016	0.942	0.001	-0.010	0.663	0.005
Model-5	0.584	0.943	0.001	0.093	0.664	0.000
Model-6	0.465	0.943	0.000	0.096	0.664	0.000
Model-7	0.826	0.943	0.000	0.391	0.666	0.002
Model-8	-0.354	0.944	0.000	0.060	0.666	0.000

* Sequential multiple regression. Model-1:

a control variables (BMI, age, waist circumference, systolic BP, Diastolic BP, duration of diabetes),

b control variables (BMI, age, systolic BP, Diastolic BP, duration of diabetes), Model-2: Model-1+HbA1c; Model-3: Model-2+FBG; Model-4: Model-3+HOMA-IR; Model-5: Model-4+CVD; Model-6: Model-5+Nephropathy; Model-7: Model-6+Neuropathy; Model-8: Model-7+Retinopathy.

*** P≤0.001

**Table-IV:** Regression analysis independent models show the predictive ability of TGI and THR for isolated biochemical variables and diabetes-associated complications

Predictors	TGI^a^			THR^b^		

	*Beta	*R^2^	*Delta R^2^	*Beta	*R^2^	*Delta R^2^
Model-1		0.201			0.232	
Model-2	4.674^***^	0.939	0.738^***^	0.737^***^	0.596	0.408^***^
Model-3	2.971^***^	0.941	0.740^***^	0.461^***^	0.582	0.395^***^
Model-4	0.097^***^	0.926	0.725^***^	0.015^***^	0.545	0.362^***^
Model-5	8.883^***^	0.555	0.354^***^	-0.132	0.329	0.170^***^
Model-6	4.118	0.247	0.046	0.575	0.161	0.020
Model-7	8.070	0.249	0.048	1.253	0.167	0.026
Model-8	-0.706	0.201	0.001	0.261	0.140	0.002

* Linear regression. Model 1:

a control variables (BMI, age, waist circumference, systolic BP, Diastolic BP, duration of diabetes),

b control variables (BMI, age, systolic BP, Diastolic BP, duration of diabetes) Model-2: Model-1+HbA1c; Model-3: Model-1+FBG; Model-4: Model-1+HOMA-IR; Model-5: Model-1+CVD; Model-6: Model-1+Nephropathy; Model-7: Model-1+Neuropathy; Model-8: Model-1+Retinopathy.

*** P≤0.001.

## DISCUSSION

In our study, high levels of TGI were found in patients with uncontrolled T2DM. Moreover, TGI also revealed a positive correlation with increased HbA1c, FBG, and HOMA-IR. In accordance, another study revealed increased TGI in patients with HbA1c greater than 7%.^11^ Furthermore, a positive correlation between TGI and uncontrolled glycemic status suggested that TGI can be used independently as a significant marker to predict the development and progression of insulin resistance (IR), T2DM and related complications.^12^,^13^ It has been demonstrated that TGI has better predictive ability than FPG for diabetes mellitus.^14^

High levels of TGI can be used as a significant variable to determine the increased risk of the development of T2DM.^8^,^15^ A strong association of higher TGI with increased CVD complications, coronary artery stenosis, non-fatal MI, stroke, and post-discharge revascularization in T2DM was found.^16^-^18^ Likewise, another study revealed a significant association of elevated TGI with retinopathy and nephropathy.^19^ Early analysis and control of TGI levels can decrease microvascular complications in patients with T2DM.^20^ On the contrary, another study demonstrated a non-significant association of TGI with retinopathy and CAD.^21^

In this study, levels of THR were also elevated in patients with uncontrolled T2DM. Likewise, THR was also positively correlated with increased HbA1c, FBG and HOMA-IR levels. In conformity, another study revealed increased THR in patients with HbA1c greater than 7%.^19^,^13^ A positive correlation between THR and uncontrolled glycemic status suggested its beneficial role in assessing glycemic control in T2DM.^22^ THR could be used as a marker for the detection of IR in obese diabetic patients.^23^ A Chinese cohort retrospective study showed that elevated THR levels had a significant positive association with the risk of development of DM.^24^

### Limitation

It is a cross-sectional single-centre study with a small number of samples. Large multicenter follow-up studies including more individuals should be planned to further evaluate the effectiveness of TGI and THR in the Pakistani population having T2DM.

## CONCLUSION

TGI and THR levels were significantly raised in T2DM patients with poor glycemic control. They can act as simple and useful markers that have the strong predictive capability to identify IR and detect the development of T2DM. Moreover, TGI can be more precisely used for prediction analysis as compared to THR.

### Author Contribution:

**WMJ:** Conceptualization, Data curation, Formal Analysis, Supervision, Writing-review, editing, accuracy and integrity of work, final approval.

**BJ:** Data curation, Methodology, Writing-original draft, formal analysis, accuracy and integrity of work.

**SK:** Methodology, Writing-review and editing.

**AA:** Writing- review and editing.
